# Functional Natural Killer-cell Genetics and Microvascular Inflammation After Kidney Transplantation: An Observational Cohort Study

**DOI:** 10.1097/TP.0000000000005228

**Published:** 2025-04-17

**Authors:** Matthias Diebold, Hannes Vietzen, Martina Schatzl, Katharina A. Mayer, Susanne Haindl, Andreas Heinzel, Philip Hittmeyer, Carsten T. Herz, Helmut Hopfer, Thomas Menter, Laura M. Kühner, Sarah M. Berger, Elisabeth Puchhammer-Stöckl, Konstantin Doberer, Jürg Steiger, Stefan Schaub, Georg A. Böhmig

**Affiliations:** 1 Division of Nephrology and Dialysis, Department of Medicine III, Medical University of Vienna, Vienna, Austria.; 2 Clinic for Transplantation Immunology and Nephrology, University Hospital Basel, Basel, Switzerland.; 3 Center for Virology, Medical University of Vienna, Vienna, Austria.; 4 Department of Pathology, Institute of Medical Genetics and Pathology, University Hospital Basel, Basel, Switzerland.

## Abstract

**Background.:**

Recent evidence highlights the pivotal role of natural killer (NK) cells in allograft rejection.

**Methods.:**

We explored associations of missing self and gene polymorphisms determining the phenotype and/or functionality of NK cells with microvascular inflammation (MVI) in a single-center cohort of 507 consecutive kidney transplant recipients. Patients were genotyped for killer cell Ig-like receptors and polymorphisms in 4 selected genes (*FCGR3A*^V/F158^ [rs396991], *KLRC2*^wt/del^, *KLRK1*^HNK/LNK^ [rs1049174], and rs9916629-C/T).

**Results.:**

MVI was detected in 69 patients (13.6%). In a proportional odds model, the *KLRC2*^del/del^ variant reduced MVI risk (odds ratio [OR] 0.26; 95% confidence interval [CI], 0.05-0.93; *P* = 0.037) independent of donor-specific antibodies, HLA class II eplet mismatch, and number of biopsies. Conversely, missing self (OR 1.40; 95% CI, 1.08-1.80; *P* = 0.011) and the rs9916629 T/T gene variant increased the risk (OR 1.70; 95% CI, 1.08-2.68; *P* = 0.021). Graft loss tended to be more frequent among patients with missing self ≥2 (hazard ratio 1.97; 95% CI, 0.89-4.37; *P* = 0.097), without influence on estimated glomerular filtration trajectories. *FCGR3A* variants were associated with MVI only in patients with preformed and/or de novo donor-specific antibodies (OR 4.14; 95% CI, 0.99-17.47; *P* = 0.052).

**Conclusions.:**

Missing self and NK-cell genetics may contribute to MVI, underscoring the important role of NK cells in transplant rejection.

## Graphical abstract

**Figure:**
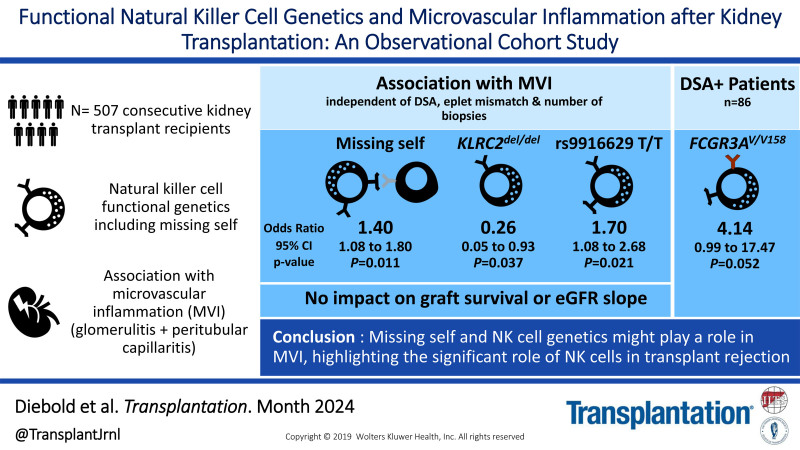

## INTRODUCTION

Antibody-mediated rejection (AMR) is a major cause of graft loss after kidney transplantation and characterized by features of microvascular inflammation (MVI), such as glomerulitis (g) and peritubular capillaritis (ptc).^1,2^ Recent studies emphasized that MVI can occur even without detectable donor-specific antibodies (DSAs), recognized in the updated Banff 2022 schema as DSA-negative and C4d-negative MVI.^3^ However, gene expression patterns remain indistinguishable, mainly triggered by natural killer (NK) cell-associated gene transcripts.^4,5^ A pivotal role of NK cells as a major driver of MVI has now been suggested by gene expression analyses as well as by studies using multiplexed immunofluorescence and spatial transcriptomics.^6-10^ In addition, there is now clinical evidence from a phase 2 trial that depletion of NK cells, via targeting CD38, leads to resolution of MVI in the context of AMR.^11^ NK-cell activation involves a complex interplay of activating and inhibitory signals mediated by a sophisticated array of receptors.^12^ One triggering factor, independent of DSA presence, could be “missing self,” where the absence of recipient HLA class I on donor cells reduces inhibitory signals to killer cell immunoglobulin-like receptors (KIRs).^13,14^ Another potential activation pathway involves the interaction between Fc gamma receptor IIIA (FcγRIIIA) and endothelium-bound DSA, supported by increased FcγRIIIA-associated transcripts in AMR.^9,10,15^ However, studies evaluating associations between a functional polymorphism in the *FCGR3A* gene (V/F_158_) and AMR occurrence have yielded mixed results.^16-20^ Recent studies have suggested the role of the C-type lectin NKG2C receptor, encoded by the *KLRC2* gene, in regulating the number and activity of NK cells.^21-23^ A polymorphism in this gene, resulting in a homozygous or heterozygous deletion of a 16-kb section, has been associated with MVI in patients with DSAs.^24,25^ The cytotoxic activity of NK cells is further influenced by polymorphisms in the *KLRK1* gene encoding the transmembrane protein NKG2D.^26^ NKG2D is activated via interaction with major histocompatibility complex class I–related chains A and B, for example, upregulated on reperfusion injury or rejection. Polymorphisms in the *KLRK1* gene have been shown to determine the cytotoxic activity of NKG2D^+^ NK cells.^27^ Finally, a recently identified polymorphism, the rs9916629-C allele, has been shown to skew NK cells toward a higher proportion of cytotoxic CD56^dim^ NK cells.^28^

This analysis, performed in a large, unselected, single-center cohort of kidney transplant recipients, aimed to explore the associations between genetically determined NK-cell functionality or subset distribution and MVI occurrence.

## MATERIALS AND METHODS

### Study Design and Patients

This prospective cohort study included 507 consecutive kidney allograft recipients transplanted at the University Hospital Basel, Switzerland, between 2015 and 2021. Clinical data and the results of routine laboratory analysis were prospectively collected during regular clinical visits. Genotyping for missing self and assessment of functional gene polymorphisms were performed retrospectively on biobanked DNA.

All patients provided written informed consent. The study was approved by the local ethics committee, and all procedures complied with the Declaration of Istanbul and the Declaration of Helsinki. We adhered to the Strengthening the Reporting of Observational Studies in Epidemiology guidelines when reporting this analysis.

### Transplant Biopsies

Overall, 459 patients had a least 1 biopsy after transplantation and the median number of biopsies per patient was 2 (interquartile range [IQR], 1–3; range, 1–7). In total, 1061 biopsies were performed. Of those, 468 were indication biopsies and 593 were protocol biopsies (142 performed after 3 mo, 143 after 6 mo, and 308 after 12 mo posttransplantation). Overall, 91% of all participants underwent protocol biopsies. Indication biopsies were performed for graft dysfunction, proteinuria, a positive DSA result, and/or, urinary chemokine (CXCL10) levels.^29^ Rejection phenotypes and morphologic single lesions, such as g, ptc, or glomerular basement membrane double contours (cg), were categorized and graded according to the 2019 Banff scheme.^30^ MVI was defined as the sum of g and ptc scores. Notably, following the Banff scheme, in the presence of thrombotic microangiopathy and de novo or recurrent glomerulonephritis, g was not scored as a single lesion. In the presence of borderline rejection, T cell–mediated rejection (TCMR), or infection, ptc was not considered for MVI scoring and AMR definition. For the latter, a g score of ≥1 was necessary. Additionally, a tubulointerstitial inflammation score (interstitial inflammation [i] plus tubulitis [t]) was calculated.

### Endpoints

The primary endpoint was the development of MVI (g+ptc score). Secondary endpoints were death-censored graft survival, patient survival, and estimated glomerular filtration rate (eGFR) slope.

### HLA Typing and Antibody Detection

Patterns of HLA reactivity were analyzed by applying single-antigen flow bead technology on a Luminex platform (LABScreen single antigen, OneLambda, West Hills CA), as described previously.^31,32^ A positive test result was defined according to a mean fluorescence intensity threshold of >500. Donor specificity of HLA antibodies was determined through virtual crossmatching based on the results of high-resolution donor/recipient HLA typing (HLA-A, -B, -C, -DR, -DQ, and/or -DP). High-resolution HLA typing was conducted on genomic DNA through next-generation sequencing using the NGSgo Workflow by GenDx (www.gendx.com). Sequencing was performed on an Illumina MiSeq instrument (www.illumina.com) using MiSeq V2 reagents. Eplet mismatches were calculated using HLAMatchmaker version 3.1.

### KIR Typing and Missing Self-calculation

KIR variants, including 2DL1, 2DL2, 2DL3, 3DL1, and 3DL2, were genotyped retrospectively on biobank DNA using the Olerup Sequence Specific Priming KIR Genotyping Kit (CareDx Inc, Brisbane, CA), as previously described.^25^ Briefly, after DNA amplification polymerase chain reaction (PCR)-sequence specific priming, electrophoresis was conducted using 2% E-Gel Agarose Gels with SYBR Safe DNA Gel Stain. The PCR products were visualized using an E-Gel Power Snap Electrophoresis Device (Thermo Fisher Scientific Inc, Waltham, MA). Missing self was defined as the absence of a corresponding HLA class I molecule in patients with educated NK cells and the specific KIR receptor.^13^ NK cells were defined as educated if recipients expressed both the specific KIR receptor within their repertoire and the corresponding HLA class I molecule (eg, 2DL1/C2, 2DL2/C1, 2DL3/C1, 3DL1/Bw4, 3DL2/HLA A3 or A11). The absence of the corresponding HLA class I molecule in the donor, combined with the presence of educated NK cells, was defined as missing self.^14^ For the C1 locus, 2 missing self types were possible if the recipient was positive for KIR 2DL2 and KIR 2DL3 and C1 and the donor did not express C1.

### Functional Gene Variants

The *FCGR3A*-V/F158 functional variants (rs396991) were examined using a QuantStudio5 real-time (PCR detection system (Applied Biosystems, Darmstadt, Germany) using TaqMan SNP Genotyping Assay and TaqMan Genotyping Master Mix.^16^ For genotyping the *KLRC2*^wt/del^ gene variants, a touchdown PCR method was used.^24^ *KLRK1*^LNK/HNK^ variants (rs1049174) were genotyped using the TaqMan SNP Genotyping Assay and TaqMan Universal PCR Master Mix (rs1049174) from Thermo Fisher Scientific, Waltham, MA. The rs9916629‐C/T polymorphism was assessed using an in-house TaqMan assay.^33^

### Statistics

Hypothesis testing used a 2-tailed approach with a *P* value of <0.05. Continuous variables are summarized as median and IQR and discrete variables are summarized as counts (percentage). Group differences were assessed using the Kruskal-Wallis test and Pearson’s chi-square test as appropriate. We used a proportional odds model, treating MVI as an ordinal variable, to examine the relationship between polymorphisms and missing self with MVI. For the model, the genetic polymorphisms were dichotomized to homozygous for the high-functional allele versus heterozygous and homozygous for the low-functional allele. For the *KLRC2* variants, we compared *KLRC2*^del/del^ to *KLRC2*^wt/del^/*KLRC2*^wt/wt^. We forced the presence of DSA (pretransplant and/or de novo) as well as the DR-, DQ-, and DP eplet mismatches and the number of biopsies per patient into the model. HLA class I mismatch was not included because of multicollinearity with missing self. The proportional odds assumption was tested with the Brant-Wald test (**Table S1, SDC,** http://links.lww.com/TP/D175). To further strengthen the results, we calculated an additional ordinal mixed-effect regression model using all biopsies with genetic variants, missing self, and time since transplantation as fixed effects, including a random intercept for patients. Death-censored graft and patient survival were assessed using the Kaplan-Meier method. Cox regression analysis was used to calculate the hazard of graft and patient survival. Cox models were adjusted for the same variables as the logistic regression model. The proportional hazard assumption was examined by inspecting the Schoenfeld residuals. The eGFR slope was estimated using a linear mixed-effects model, starting 3 mo posttransplantation, including a knot at 1 y. No imputation was used for missing values. Statistical analyses were performed using R software, version R 4.0.2 (R Core Team 2020. R: a language and environment for statistical computing; R Foundation for Statistical Computing, https://www.R-project.org). The packages used for this analysis are provided as **List of R Packages Used** (**SDC,** http://links.lww.com/TP/D175). For the graphical representation, Biorender.com was used.

## RESULTS

### Patients

**FIGURE 1.**
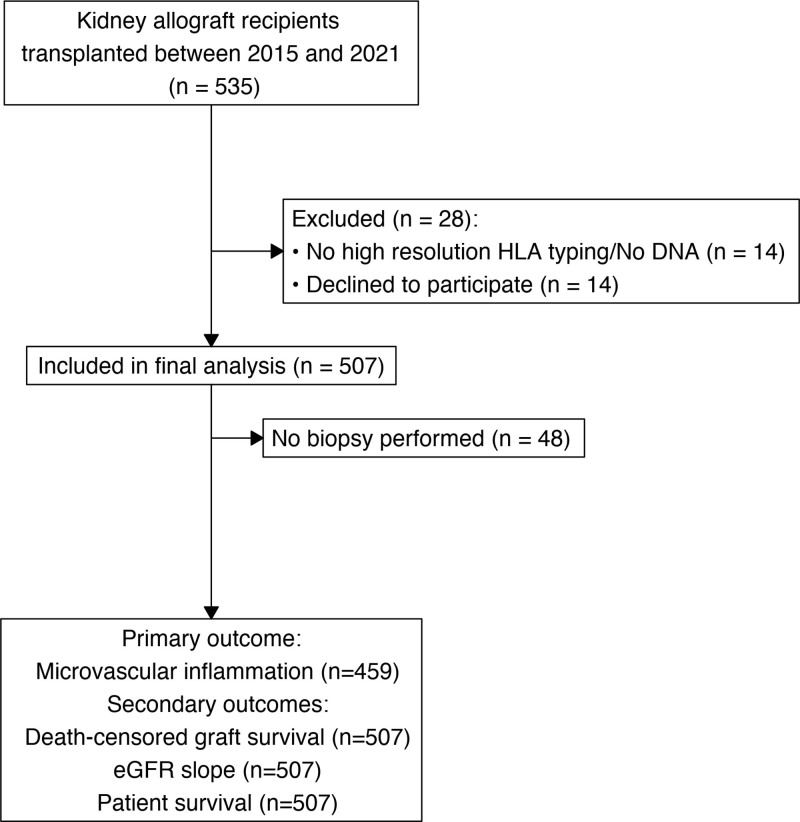
Study flowchart. Between 2015 and 2021, 535 patients underwent kidney transplantation at the University Hospital Basel. Out of these, 507 were included in the study. Forty-eight patients who did not undergo biopsies were excluded from the primary endpoint analysis (microvascular inflammation). eGFR, estimated glomerular filtration rate.

In this single-center prospective cohort study, a total of 507 kidney allograft patients were included (Figure 1). Baseline characteristics, stratified to the presence of MVI, are detailed in Table 1. The cohort comprised 189 female patients (37.3%), with a median age at transplantation of 55 (IQR, 45–63) y. Two hundred nine patients (41.2%) were recipients of a living donor transplant, and 58 patients (11.4%) had preformed HLA-DSAs. Induction therapy predominantly included basiliximab (77.9%); 81 patients (16%) received antithymocyte globulin and 31 (6.1%) did not receive any induction therapy. Most patients received tacrolimus-based triple immunosuppression. Patients with MVI more often had preformed DSAs (*P* = 0.048), a higher eplet mismatch in HLA-A, -B, and -C (*P* = 0.037), and higher levels of calculated panel reactivity (*P* = 0.001). Consequently, patients with MVI more often received antithymocyte globulin and less often basiliximab as induction therapy (*P* = 0.023; Table 1).

**TABLE 1.** Baseline characteristics of the study population in relation to MVI

Baseline characteristics	Total (N = 507)	No MVI^*a*^ (N = 390)	MVI^*a*^ (N = 69)	*P*
Female recipient sex, n (%)	189 (37.3)	133 (34.1)	33 (47.8)	0.040
Recipient age, y, median (IQR)	55 (45–63)	55 (45–63)	52 (44–63)	0.346
Donor type, n (%)				1.000
Deceased donor	298 (58.8)	230 (59.0)	41 (59.4)	
Living donor	209 (41.2)	160 (41.0)	28 (40.6)	
Donor age, y, median (IQR)	57 (48–65)	57 (48–64)	55 (49–65)	0.852
CMV risk constellation, n (%)				
D^+^/R^+^	175 (34.5)	127 (32.6)	32 (46.4)	0.192
D^–^/R^+^	117 (23.1)	90 (23.1)	16 (23.2)	
D^–^/R^–^	117 (23.1)	97 (24.9)	11 (15.9)	
D^+^/R^–^	97 (19.1)	75 (19.2)	10 (14.5)	
Unknown	1 (0.2)	1 (0.3)	0 (0.0)	
Cold ischemia time, min, median (IQR)	421 (114–603)	420 (115–600)	415 (104–675)	0.863
First transplant, n (%)	405 (88.2)	347 (89.0)	58 (84.1)	0.334
Risk category, n (%)				0.048
Standard risk	399 (78.7)	317 (81.3)	45 (65.2)	
Immunological risk	108 (21.3)	73 (18.7)	24 (34.8)	
HLA-DSA	56 (11.0)	36 (9.2)	13 (18.8)	
ABOi	34 (6.7)	25 (6.4)	8 (11.6)	
HLA-DSA plus ABOi	4 (0.8)	3 (0.8)	1 (1.4)	
Other^*b*^	14 (2.8)	9 (2.3)	2 (2.9)	
HLA mismatch in A, B, DR, median (IQR)	4 (3–5)	4 (3–5)	5 (4–5)	0.094
Eplet mismatch in				
A, B, C, median (IQR)	10 (7–14)	10 (7–14)	12 (10–14)	0.037
DR, median (IQR)	4 (2 to 7)	5 (2 to 7)	4 (2 to 6)	0.374
DQ, median (IQR)	4 (1 to 6)	4 (1 to 6)	4 (1 to 6)	0.979
DP, median (IQR)	1 (0 to 3)	1 (0 to 3)	2 (0 to 3)	0.632
DSA specificity, n (%)				0.756
HLA class I only	19 (32.8)	12 (32.4)	4 (28.6)	
HLA class II only	27 (46.6)	18 (48.6)	6 (42.9)	
HLA class I + II	12 (20.7)	7 (18.9)	4 (28.6)	
MFI of immunodominant DSA, median (IQR)	1316 (526–2214)	138 (689–2331)	904 (569–1447)	0.308
Current cPRA, median (IQR)	6.9 (0.0–53.1)	5.5 (0.0–45.6)	31.8 (0.6–72.6)	0.001
Induction therapy, n (%)				0.023
Basiliximab	395 (77.9)	314 (80.5)	49 (71.0)	
ATG	81 (16.0)	53 (13.6)	18 (26.1)	
None	31 (6.1)	23 (5.9)	2 (2.9)	
Maintenance immunosuppression, n (%)				0.911
Tac-MMF-steroid	333 (65.7)	258 (66.2)	46 (66.7)	
Tac-MPS-steroid	170 (33.5)	129 (33.1)	23 (33.3)	
Tac-Aza-steroid	2 (0.4)	2 (0.5)	0 (0.0)	
CyA-MMF-steroid	2 (0.4)	1 (0.3)	0 (0.0)	

Values are presented as n (%) if not otherwise stated.

a No biopsies were performed in 48 patients, therefore, calculation of MVI was only possible in 459 patients.

b Other risks include husband to wife with shared children transplantation, child to mother, and repeated mismatches from prior transplantations.

ATG, antithymocyte globulin; Aza, azathioprine; CMV, cytomegalovirus; cPRA, calculated panel-reactive antibody; CyA, cyclosporine A; DSA, donor-specific antibody; IQR, interquartile range; MFI, mean fluorescence intensity; MMF, mycophenolate mofetil; MPS, mycophenolate sodium; MVI, microvascular inflammation; Tac, tacrolimus.

### Genotyping Results

**FIGURE 2.**
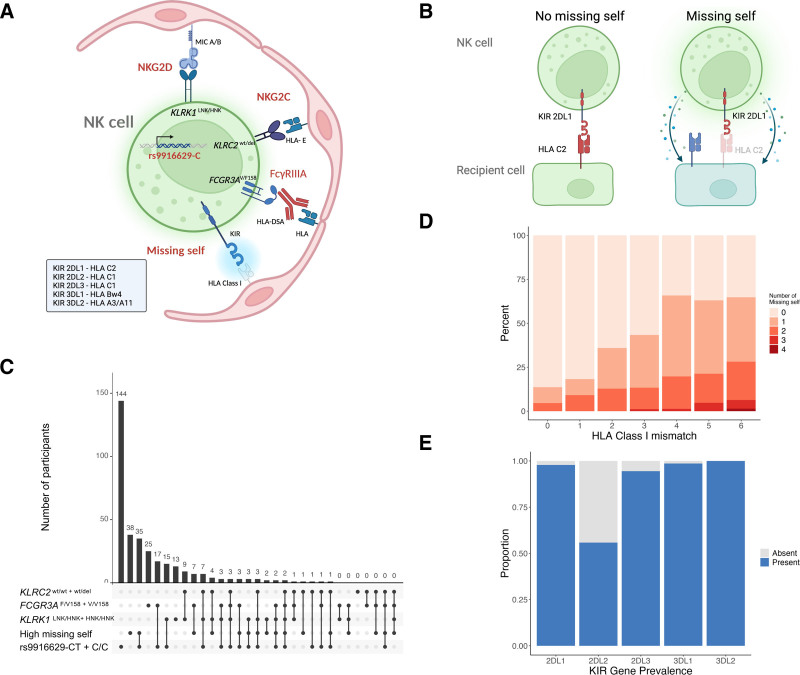
Genetic background of NK-cell activation in the microcirculation. A, The schematic illustration of NK activation pathways potentially contributing to microcirculation inflammation. These include NK-cell activation via interaction of MHC class I polypeptide-related sequence MIC A/B with NKG2D, NKG2c with HLA-E, Fc gamma receptor IIIA FcγRIIIA with donor-specific antibodies bound to HLA and the NK cells associated rs9916629‐C allele. Furthermore, the mechanism of missing self is shown, depicting a KIR and the absence of the corresponding HLA class I. The missing self-types are shown in the gray box. B, The mechanism of missing self. KIRs bind to corresponding HLA class I molecules, as depicted in the gray box (A). The binding of KIR with its corresponding HLA class I molecule leads to the inactivation of the NK cell. The absence of the corresponding HLA class I molecule results in missing self and the activation of NK cells. C, Delineates observed and possible combinations between the distinct genetic variants and missing self. Combinations are depicted using black dots and connected lines, with the upper part of the graph indicating the number of participants for individual combinations. D, The correlation between HLA class I mismatch (HLA-A + HLA-B + HLA-C) and missing self. The panel illustrates the detection of missing self in some of the HLA class I-matched donor-recipient pairs (eg, donor expressed only C1, recipient expresses C1 and C2). E, The prevalence of KIR genes relevant for missing self-calculation within the studied kidney transplant cohort. DSA, donor-specific antibody; KIR, killer cell immunoglobulin-like receptor; NK, natural killer cell.

Figure 2A and B shows the NK cell activation pathways that may contribute to microcirculation inflammation. Figure 2C represents the interplay between individual genotypes and the missing self, showcasing both observed occurrences and potential combinations. Missing self was identified in 280 patients (55.1%), and 179 patients (35.2%) had 1, 84 (16.5%) had 2, 15 (3.0%) had 3, and 2 (0.4%) had 4 missing self types, respectively. Missing self for HLA-A3/A11 was found in 96 patients (18.9%), for HLA-Bw4 in 110 patients (21.7%), for HLA-C1 (KIR 2DL2) in 33 patients (6.5%), HLA-C1 (KIR 2DL3) in 54 patients (10.6%), and for HLA-C2 in 107 patients (21.1%), respectively. Missing self correlated with the level of HLA class I mismatch (Spearmans ρ: 0.25, *P* < 0.001). Table 2 depicts the distribution of the different KIR genes, missing self, predicted missing self calculated without KIR typing results, and HLA class I mismatches. The correlations between HLA class I mismatch and missing self are shown in Figure 2D, and the prevalence of KIR receptors is shown in Figure 2E.

**TABLE 2.** Donor and recipient genetic background in relation to MVI

	Total	No MVI^*a*^	MVI^*a*^
KIR typing			
KIR2DL1	496 (97.8)	384 (98.5)	65 (94.2)
KIR2DL2	283 (55.8)	217 (55.6)	41 (59.4)
KIR2DL3	479 (94.5)	372 (95.4)	64 (92.8)
KIR3DL1	500 (98.6)	385 (98.7)	68 (98.6)
KIR3DL2	507 (100)	390 (100)	69 (100)
Missing self			
HLA-A3/A11	96 (18.9)	77 (19.7)	13 (18.8)
HLA-Bw4	110 (21.7)	82 (21.0)	19 (27.5)
HLA-C1 - (KIR2DL2)	33 (6.5)	24 (6.2)	6 (8.7)
HLA-C1 - (KIR2DL3)	54 (10.6)	43 (11.0)	7 (10.1)
HLA-C2	107 (21.1)	74 (19.0)	21 (30.4)
Predicted missing self			
pHLA-A3/A11	96 (18.9)	77 (19.7)	13 (18.8)
pHLA-Bw4	112 (22.0)	83 (21.3)	19 (27.5)
pHLA-C1 - (KIR2DL2)	57 (11.2)	44 (11.3)	8 (11.6)
pHLA-C1 - (KIR2DL3)	57 (11.2)	44 (11.3)	8 (11.6)
pHLA-C2	111 (21.9)	76 (19.5)	22 (31.9)
HLA class I mismatch			
HLA-A mismatch			
0	68 (13.4)	51 (13.1)	2 (2.9)
1	221 (43.5)	171 (43.8)	34 (49.3)
2	219 (43.1)	168 (43.1)	33 (47.8)
HLA-B mismatch			
0	37 (7.3)	27 (6.9)	0 (0.0)
1	165 (32.5)	133 (34.1)	22 (31.9)
2	306 (60.2)	230 (59.0)	47 (68.1)
HLA-C mismatch			
0	49 (9.6)	35 (9.0)	4 (5.8)
1	200 (39.4)	154 (39.5)	29 (42.0)
2	259 (51.0)	201 (51.5)	36 (52.2)

a An MVI score ≥2.

KIR, killer-cell immunoglobulin-like receptor; MVI, microvascular inflammation.

Two hundred nine participants (41.2%) were homozygous for the low functional allele (*FCGR3A*^F/F158^), 234 (46.2%) were heterozygous (*FCGR3A*^V/F158^), and 64 patients (12.6%) were homozygous for the high-affinity allele (*FCGR3A*^V/V158^). Most patients were genotyped for the *KLRC2*^wt/wt^ variant (n = 343, 67.8%). *KLRC2*^wt/del^ was identified in 130 patients (25.7%) and *KLRC2*^del/del^ in 33 patients (6.5%). The *KLRK1*^LNK/LNK^ polymorphism was present in 251 patients (49.5%), whereas 189 patients (37.3%) and 67 patients (13.2%) encoded for the *KLRK1*^LNK/HNK^ and *KLRK1*^HNK/HNK^ genotype, respectively. The rs9916629-C/C polymorphism was detected in 51 patients (10.1%), 215 patients (42.4%) had rs9916629‐C/T and 241 (47.5%) rs9916629‐T/T genotypes. The distribution of *KLRK1* and *KLRC2* variants in our cohort deviated from the Hardy-Weinberg equilibrium (**Table S2, SDC,** http://links.lww.com/TP/D175). We found a strong correlation between *KLRC2* and *KLRK1* genotypes (Spearmans ρ: 0.70, *P* < 0.001). No other correlations were observed, including missing self versus individual genetic variants (**Figure S1, SDC**, http://links.lww.com/TP/D175).

### NK-cell Genetics and MVI

**FIGURE 3.**
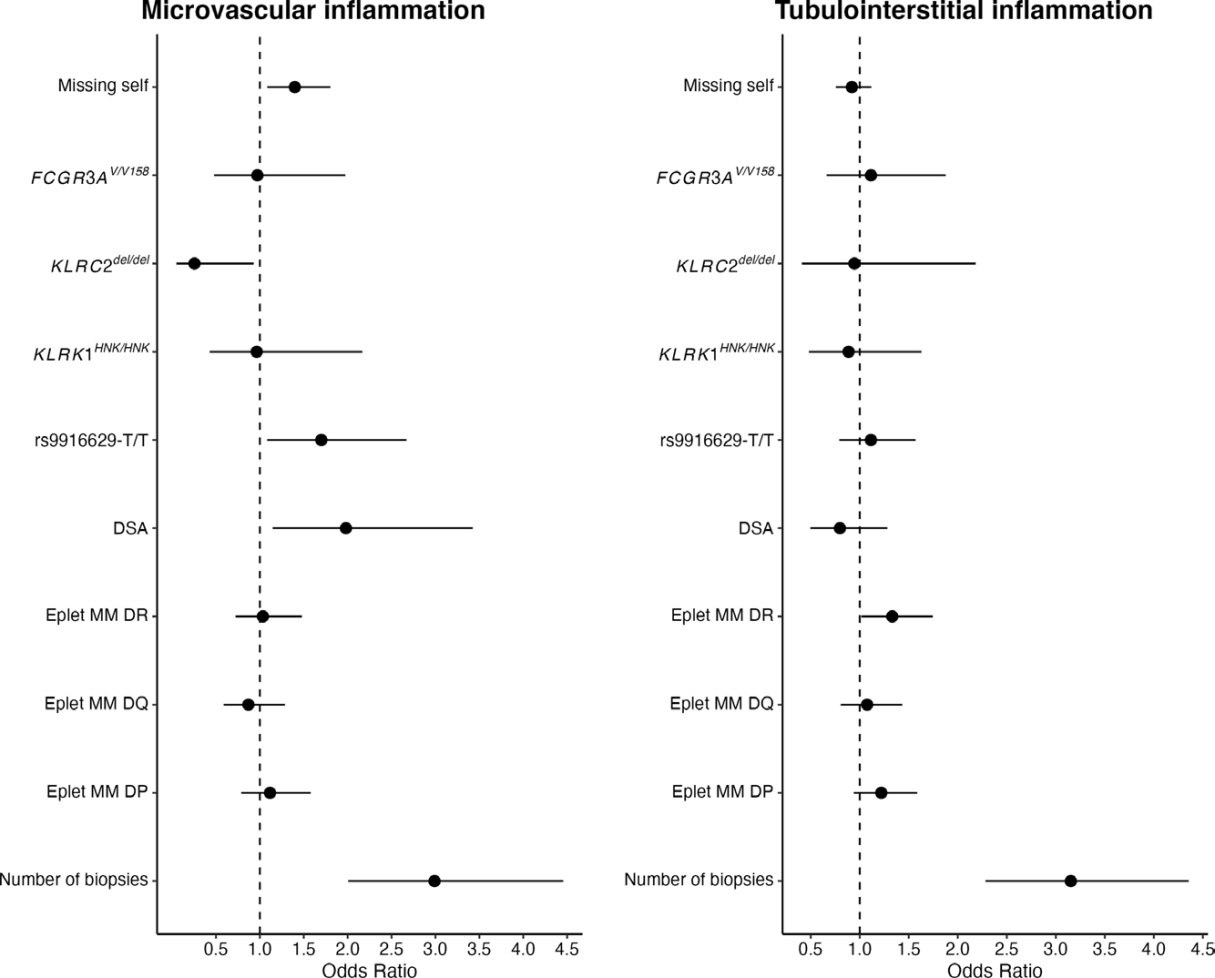
Natural killer cell genetics in relation to morphologic results. The results of proportional odds regression models are shown for microvascular inflammation (g+ptc score; A) and the extent of tubulointerstitial inflammation (t + i score; B). Both models included a donor-specific antibody result and eplet mismatch in HLA class II as forced variables; hence, the estimates should be interpreted with caution.

A total of 1061 biopsies were performed, with a median of 2 (IQR, 1–3) biopsies per patient. Forty-eight patients (9.6%) had no biopsy and were not included in the MVI analysis. MVI was detected in 199 biopsies (18.8%) corresponding to 69 patients (15%). The distribution of the genetic variants and missing self in relation to MVI is shown in **Figure S2** (**SDC,** http://links.lww.com/TP/D175). Notably, no patients with *KLRC2*^del/del^ showed MVI (g+ptc ≥2) in their biopsies. In univariable analysis, the number of missing self was associated with higher odds (odds ratio [OR] 1.30; 95% confidence interval [CI], 1.02-1.66; *P* = 0.035) and the *KLRC2*^del/del^ variant with lower odds (OR 0.32; 95% CI, 0.08-0.93; *P* = 0.035) of MVI. A nonstatistically significant association was found for the rs9916629‐T/C variant (OR 1.46; 95% CI, 0.96-2.25; *P* = 0.079; Table 3). No associations were found for *KLRK1* or *FCGR3A* variants. In a multivariable model, adjusted for the presence of de novo/preformed DSA, eplet mismatches, and the number of biopsies, missing self (OR 1.40; 95% CI, 1.08-1.80; *P* = 0.011), *KLRC2*^del/del^ (OR 0.26; 95% CI, 0.05-0.93; *P* = 0.037), and rs9916629‐T/C variant (OR 1.70; 95% CI, 1.08-2.68; *P* = 0.021) were significantly associated with MVI (Figure 3A). No association was found between missing self or single gene variants and the extent of tubulointerstitial inflammation (i+t score; Figure 3B). Similar results were seen in a mixed-effect ordinal regression model, including the MVI results obtained in all biopsies, without reaching the conventional boundaries of statistical significance (**Table S3, SDC,** http://links.lww.com/TP/D175).

**TABLE 3.** Proportional odds regression model for MVI^*a*^

Variants	Univariable		Multivariable^*b*^	
	OR (95% CI)	*P*	OR (95% CI)	*P*
Missing self	1.3 (1.02-1.66)	0.035	1.40 (1.08-1.80)	0.011
*FCGR3A* ^V/V158^	0.99 (0.49-1.86)	0.974	0.97 (0.46-1.92)	0.937
*KLRC2* ^del/del^	0.32 (0.08-0.93)	0.035	0.26 (0.05-0.93)	0.037
*KLRK1* ^HNK/HNK^	0.80 (0.40-1.53)	0.527	0.96 (0.41-2.11)	0.929
rs9916629 T/T	1.46 (0.96-2.25)	0.079	1.70 (1.08-2.68)	0.021
DSA	2.03 (1.2-3.39)	0.009	1.98 (1.13-3.4)	0.017
Eplet mismatch (DR)	1.02 (0.96-1.08)	0.615	1.01 (0.94-1.08)	0.852
Eplet mismatch (DQ)	1.01 (0.94-1.08)	0.808	0.97 (0.9-1.05)	0.482
Eplet mismatch (DP)	1.04 (0.93-1.16)	0.461	1.04 (0.92-1.16)	0.538
No. of biopsies	1.68 (1.39-2.04)	<0.001	1.73 (1.42-2.12)	<0.001

a Microvascular inflammation is used as an ordinal variable.

b DSA positivity and eplet mismatch and the number of biopsies were forced in the model; therefore, caution should be used in the interpretation of the multivariable estimates.

CI, confidence interval; DSA, donor-specific antibody; MVI, microvascular inflammation; OR, odds ratio.

### NK Genetics and Graft Survival

**FIGURE 4.**
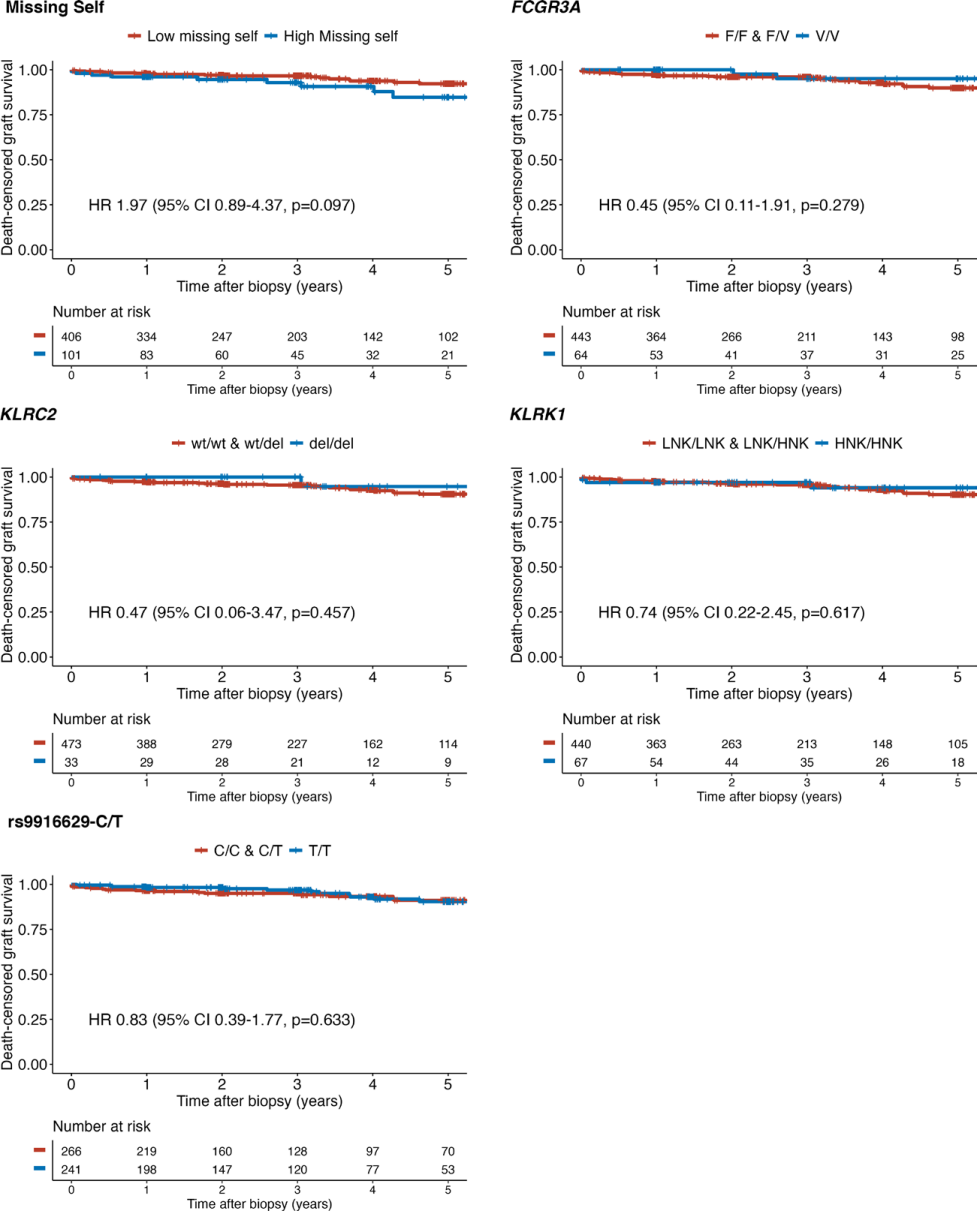
Graft survival in relation to functional natural killer cell genetics. Kaplan-Meier death-censored graft survival was analyzed in relation to missing self, *FCGR3A*, *KLRC2*, *KLRK1*, and rs9916629-C/T genotypes. The Cox regression model was adjusted for the presence of donor-specific antibodies and eplet mismatch in HLA class II. For survival analyses, missing self was dichotomized into high (≥2) and low missing self-types (<2). Individual single-gene polymorphisms were dichotomized as described in the methods section. CI, confidence interval; HR, hazard ratio.

The median follow-up time of all patients was 3 y (IQR, 1–5). Twenty-eight patients (5.5%) lost their graft, resulting in a 94.5% 5-y death-censored graft survival. Only the presence of a high missing self type (≥2) showed a statistically nonsignificant difference in death-censored graft survival (hazard ratio 1.97; 95% CI, 0.89-4.37; *P* = 0.097; Figure 4). Notably, of the 10 patients with high missing self who lost the graft, only 3 (30%) displayed MVI in prior transplant biopsies. Individual causes of graft loss are detailed in **Table S4** (**SDC,** http://links.lww.com/TP/D175). No associations were found for patient survival (**Figure S3, SDC,** http://links.lww.com/TP/D175). Similarly, there were no significant differences with respect to eGFR trajectories during the first year, after the first year, and total (**Table S5, SDC,** http://links.lww.com/TP/D175).

### Sensitivity Analyses

In a sensitivity analysis, we restricted the analysis to DSA-positive patients. Of the overall cohort, 58 patients had preformed DSAs and 30 patients developed DSAs during the observation period, resulting in 86 patients with either preformed and or de novo DSA. Among these, 22 (25.6%) developed MVI. In the proportional odds model, we found associations between *FCGR3A* (OR 4.14, 95% CI 0.99 to 17.47, *P* = 0.052) and rs9916629‐T/C variant (OR 2.56; 95% CI, 1.02-6.66; *P* = 0.046) and MVI (**Table S6, SDC,** http://links.lww.com/TP/D175). No significant associations were found for the other variants or missing self. We further investigated factors that could potentially influence NK-cell activation, such as cytomegalovirus infection, donor type, and cold ischemia time. Presumably limited by the small sample size, however, we did not observe any substantial differences in relation to these variables (**Figures S4–S6, SDC,** http://links.lww.com/TP/D175).

Finally, we repeated the analyses using predicted missing self without the inclusion of KIR typing results. The results for predicted missing self were comparable to the main analysis, showing consistent findings in both univariable (OR 1.24; 95% CI, 0.98-1.55; *P* = 0.071) and multivariable models (OR 1.32; 95% CI, 1.03-1.68; *P* = 0.026).

## DISCUSSION

This study investigated the impact of a predefined set of distinct genetic polymorphisms known to determine NK-cell functionality and subset distribution, as well as missing self, on the occurrence of MVI in a large single-center cohort of kidney transplant recipients. Our major findings highlight significant associations of missing self, as well as a polymorphism in the *KLRC2* gene and an rs9916629-T/C variant, with MVI, independently of the presence of DSA, eplet mismatch in HLA class II, and number of biopsies. Additionally, we found associations for a high-affinity *FCGR3A* gene variant, although this effect was only observed in a sensitivity analysis including DSA-positive patients. The results reinforce an important involvement of NK cells in both DSA-dependent and DSA-independent alloimmune responses.

Previous studies on the role of missing self in kidney transplantation have yielded mixed results. Although some studies reported no association between the number of missing self and graft survival or rejection episodes,^34,35^ others found significant outcome effects.^13,14,36,37^ However, as previously highlighted,^38^ discrepancies in methodology and chosen endpoints may partially explain this controversy. In a previous analysis of DSA-positive patients, we did not observe any association between the number of missing self and the occurrence of MVI.^25^ However, these results are not necessarily contradictory to our current results but may be attributed to dominant alternative pathways of NK-cell activation in this specific context, such as FcγRIIIA-triggered activation of NKG2C^+^ NK cells. Furthermore, in a sensitivity analysis focusing on DSA-positive transplant recipients, the significant effect of missing self-observed in the unselected cohort was lost, and an effect of the high-affinity FCGR3A gene variant became evident.

Recent studies suggested that missing self could account for cases of DSA-negative MVI,^4,13,38,39^ which is especially pertinent in light of the redefined MVI categories in the Banff 2022 scheme.^3^ However, accurately differentiating between DSA-positive and DSA-negative MVI remains challenging. Factors such as antibody absorption in the graft or local HLA-DSA production could have influenced our study results. Furthermore, not all patients in our cohort underwent DSA measurement at the time of biopsy, and the lack of non-HLA antibody results raises the possibility of underestimating the number of DSA-positive participants.^38,40^ Despite these limitations, our analysis revealed a similar OR in the univariable and DSA-adjusted analysis, suggesting a robust effect of missing self on MVI.

Although we identified an association of missing self with death-censored graft survival, a careful interpretation is warranted. On assessing the causes of individual graft losses, we found no consistent diagnosis, and only a few patients actually exhibited MVI, one of them classified as having no AMR, but fulfilling the criteria of C4d-negative DSA-negative MVI according to the updated Banff 2022 scheme.^3^ This suggests that our finding might be because of chance, as supported by the lack of difference in eGFR slopes. Interestingly, we were able to replicate the results of the association between missing self and MVI in a sensitivity analysis independent of KIR typing. This consistency can be attributed to the high prevalence of the KIR involved in missing self in our predominantly European transplant cohort.

Consistent with the hypothesis that other activating mechanisms influencing NK cells may contribute to MVI, we identified associations for 2 distinct functional single-gene polymorphisms. Our results validated the association between a deletion in the *KLRC2* gene and MVI. However, in contrast to a previous study, performed specifically in DSA-positive transplant recipients, our larger cohort included a considerable number of patients with a homozygous deletion, suggested to associate with the complete absence of highly cytotoxic NKG2C^+^ cells.^21-23^ None of those patients developed high MVI (eg, >1). Although this finding holds potential, its generalizability needs validation in a larger cohort. The exact pathophysiological mechanism through which NKG2C^+^ cells contribute to MVI remains unclear. Speculation includes activation via viral or human peptides that are bound to HLA-E, which is upregulated in an inflammatory state.^24,41^ However, our analyses, including a comprehensive study of the Collaborative Transplant Study cohort, found no differences in survival or eGFR slope between KLRC2 variants.^24^ The reasons for unaffected clinical outcomes remain speculative. One possible explanation could be the comparatively high 5-y death-censored graft survival rate in our cohort, and the sample size may have been insufficient to detect subtle differences in this context.^42^

Next, we identified an association between rs9916629‐C/T polymorphism and MVI, but again, without any effects on graft survival or eGFR slope. The rs9916629‐C allele has previously been shown to influence the distribution of NK-cell subsets, promoting a higher proportion of regulatory CD56^bright^ and a lower number of cytotoxic CD56^dim^ cells in addition to a reduction in the overall frequency of circulating NK cells.^28^ This redistribution was suggested to result from alterations in the expression of initial transcription factors necessary for the development of NK cells.^28^ Notably, the same polymorphism has previously been linked to fatal COVID-19 infections.^33^

Several studies have highlighted an important role of FcγRIIIA in AMR, primarily by demonstrating increased FcγRIIIA transcripts in this specific context.^10,15^ It was hypothesized that the binding of FcγRIIIA to alloreactive DSAs of IgG type bound to the graft endothelium is an important contributor to deleterious NK-cell activation. A recent analysis found an association between a high-affinity FCGR3A gene variant and MVI in DSA-positive patients.^16,20,25^ Notably, in the present analysis, this effect was observed primarily in a sensitivity analysis of DSA-positive patients.

Overall, our results align with the hypothesis that NK cells can be activated through various pathways, implicating their varied involvement in rejection processes. Although activation in the presence of DSA may primarily occur through the FcγRIIIA receptor, dependent on the affinity influenced by *FCGR3A* variants, other activation factors of NK cells may come into play in the absence of DSA. Importantly, we observed no interactions between functional gene variants and missing self. This suggests that involved activating mechanisms may act independently, emphasizing the complexity of NK-cell activation. It was previously suggested that missing self alone may not be sufficient for NK-cell activation; rather, NK cells must be primed beforehand by viral infections (eg, cytomegalovirus infection) or ischemia.^14^ In the present study, we did not find a strong link between such priming factors and the impact of NK-cell genetics or missing self on MVI. However, limitations in sample size underscore the need for larger (multicenter) cohorts to specifically address this question.

Despite using several strategies to mitigate the risk of bias and confounding, the study has limitations. First, residual confounding is likely because of the cohort study design. However, combining prospective data collection with baseline data measurements preceding the outcomes may have ensured temporality and reduced the risk of selection bias. Furthermore, although most patients underwent protocol biopsies, several also underwent diagnostic biopsies. The number and timing of these biopsies might have influenced the detection of MVI. To address this, we adjusted our model by incorporating the number of biopsies obtained. We made this decision to use the most parsimonious model because of the low number of biopsies per patient.^43^ Nevertheless, when modeling the data in an additional mixed-effects regression model that incorporated a random patient intercept and posttransplant time, we obtained results with similar point estimates. Second, the distribution of the *KLRK1* and *KLRC2* variants deviated from the Hardy-Weinberg equilibrium. Although the deviation regarding the *KLRC2* gene can be attributed to the mechanism of a deletion,^44^ we have no explanation for the deviation regarding *KLRK1* gene variants and the lower proportion of patients with *KLRK1*^LNK/LNK^ compared with previous analyses.^25^ Third, our study is a single-center analysis predominantly involving Europeans, limiting the generalizability of our findings. This is important because the distribution of KIR genes and functional gene variants might be different in other ethnic groups.^45^ Finally, the limited number of patients and events in our single-center study calls for a cautious interpretation of the data and underscores the need for further confirmatory studies.

In conclusion, our study highlights the substantial impact of genetic factors related to NK-cell functionality, specifically missing self, polymorphisms in *KLRC2* rs9916629‐C/T, and, in DSA-positive patients, *FCGR3A*, on the risk of MVI occurrence in kidney transplant recipients. These results emphasize the crucial role of NK cells in kidney transplant rejection. Furthermore, our findings suggest that targeted therapeutic approaches focusing on NK cells may hold promise in mitigating MVI-associated graft injury.

## ACKNOWLEDGMENTS

The authors thank Tobias Zimmermann and Alexander Kainz for their help with the statistical analysis.

## Supplementary Material


