# Supplementary material for: Functional Natural Killer-cell Genetics and Microvascular Inflammation After Kidney Transplantation: An Observational Cohort Study
Source: Transplantation. 2025 Apr 17;109(5):860–70. doi: 10.1097/TP.0000000000005228 (PMC12011434; doi:10.1097/TP.0000000000005228)
Supplement: Supplementary file 1 [file tpa-109-860-s001.pdf]

Missing Self and Natural Killer Functional Cells Genetics and Microvascular Inflammation – a prospective cohort study

Matthias Diebold, Hannes Vietzen, Martina Schatzl, Katharina A. Mayer, Susanne Haindl, Andreas Heinzel, Philip Hittmeyer, Carsten T. Herz, Helmut Hopfer, Thomas Menter, Laura M. Kühner, Sarah M. Berger, Elisabeth Puchhammer-Stöckl, Konstantin Doberer, Jürg Steiger, Stefan Schaub, Georg A. Böhmig

Table of contents

LIST OF R PACKAGES USED 2
FIGURE S1 CORRELATION HEATMAP. 3
FIGURE S2: DISTRIBUTION OF GENETIC VARIANTS, MISSING SELF IN RELATION TO MVI. 4
FIGURE S3: KAPLAN-MEIER PATIENT SURVIVAL IN RELATION TO MISSING SELF AND SINGLE GENE VARIANTS. 5
FIGURE S4: DISTRIBUTION OF MVI AMONG GENETIC VARIANTS AND MISSING SELF, STRATIFIED BY CMV VIREMIA 6
FIGURE S5: DISTRIBUTION OF MVI AMONG GENETIC VARIANTS AND MISSING SELF, STRATIFIED BY DONOR TYPE 8
FIGURE S6 DISTRIBUTION OF MVI AMONG GENETIC VARIANTS AND MISSING SELF, STRATIFIED BY 9
COLD ISCHEMIA TIME 9

List of R packages used

- **Tidyverse:** Wickham H, Averick M, Bryan J, Chang W, McGowan LD, François R, Grolemund G, Hayes A, Henry L, Hester J, Kuhn M, Pedersen TL, Miller E, Bache SM, Müller K, Ooms J, Robinson D, Seidel DP, Spinu V, Takahashi K, Vaughan D, Wilke C, Woo K, Yutani H (2019). "Welcome to the tidyverse." _Journal of Open Source Software_, *4*(43), 1686. doi:10.21105/joss.01686 <https://doi.org/10.21105/joss.01686>.
- **Finalfit:** Harrison E, Drake T, Ots R (2023). _finalfit: Quickly Create Elegant Regression Results Tables and Plots when Modelling_. R package version 1.0.6, <https://CRAN.R-project.org/package=finalfit>.
- **MASS:** Venables, W. N. & Ripley, B. D. (2002) Modern Applied Statistics with S. Fourth Edition. Springer, New York. ISBN 0-387-95457-0
- **Lme4:** Douglas Bates, Martin Maechler, Ben Bolker, Steve Walker (2015). Fitting Linear Mixed-Effects Models using lme4. Journal of Statistical Software, 67(1), 1-48. doi:10.18637/jss.v067.i01.
- **lmerTest**: Kuznetsova A, Brockhoff PB, Christensen RHB (2017). "lmerTest Package: Tests in Linear Mixed Effects Models." _Journal of Statistical Software_, *82*(13), 1-26. doi:10.18637/jss.v082.i13 <https://doi.org/10.18637/jss.v082.i13>.
- **UpsetR**: Conway, J. R., Lex, A., & Gehlenborg, N. (2017). UpSetR: an R package for the visualization of intersecting sets and their properties. Bioinformatics (Oxford, England), 33(18), 2938–2940. https://doi.org/10.1093/bioinformatics/btx364
- **ggplot2**: Wickham H (2016). *ggplot2: Elegant Graphics for Data Analysis*. Springer-Verlag New York. ISBN 978-3-319-24277-4, https://ggplot2.tidyverse.org.
- **ordinal**: Christensen R (2023). ordinal—Regression Models for Ordinal Data. R package version 2023.12-4, https://CRAN.R-project.org/package=ordinal.

Figure S1 Correlation Heatmap.

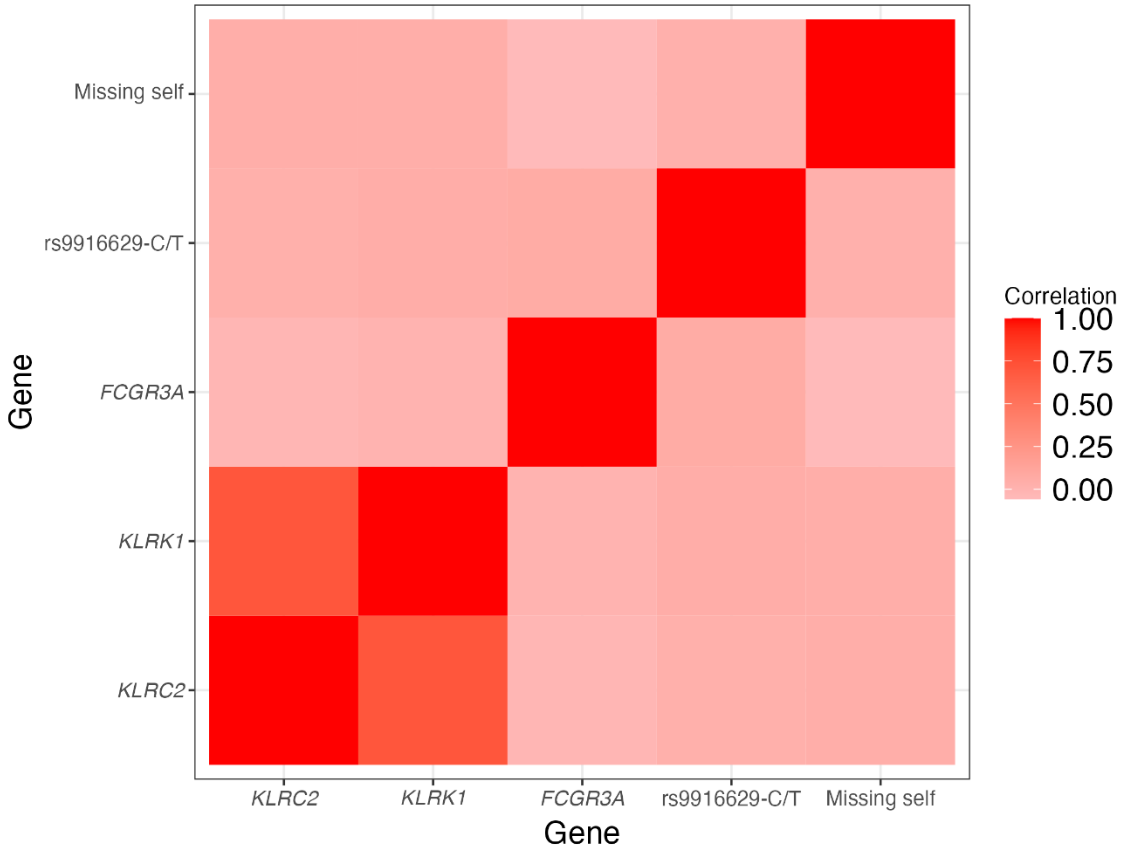

Correlation Heatmap to visualize the strength of associations between distinct genetic variants and missing self, respectively. Cells are color-coded based on the calculated Spearman´s correlation coefficients for each pair of variables.

Figure S2: Distribution of genetic variants, missing self in relation to MVI.

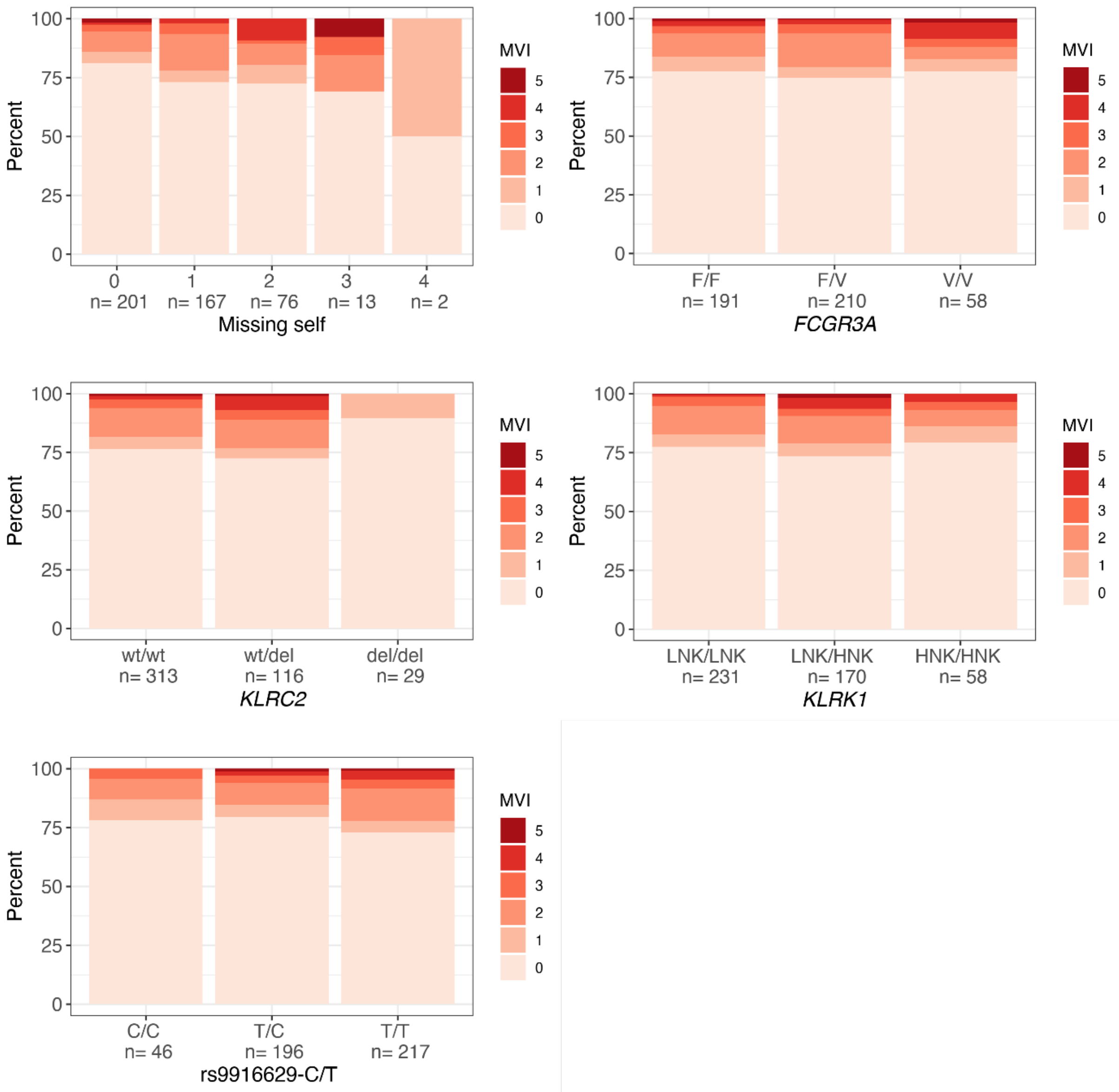

The bars represent subjects stratified to the individual genotypes or number of missing selfs, respectively. Proportions of individual MVI scores are indicated by different colors of red. Abbreviations: MVI, microvascular inflammation.

Figure S3: Kaplan-Meier patient survival in relation to missing self and single gene variants.

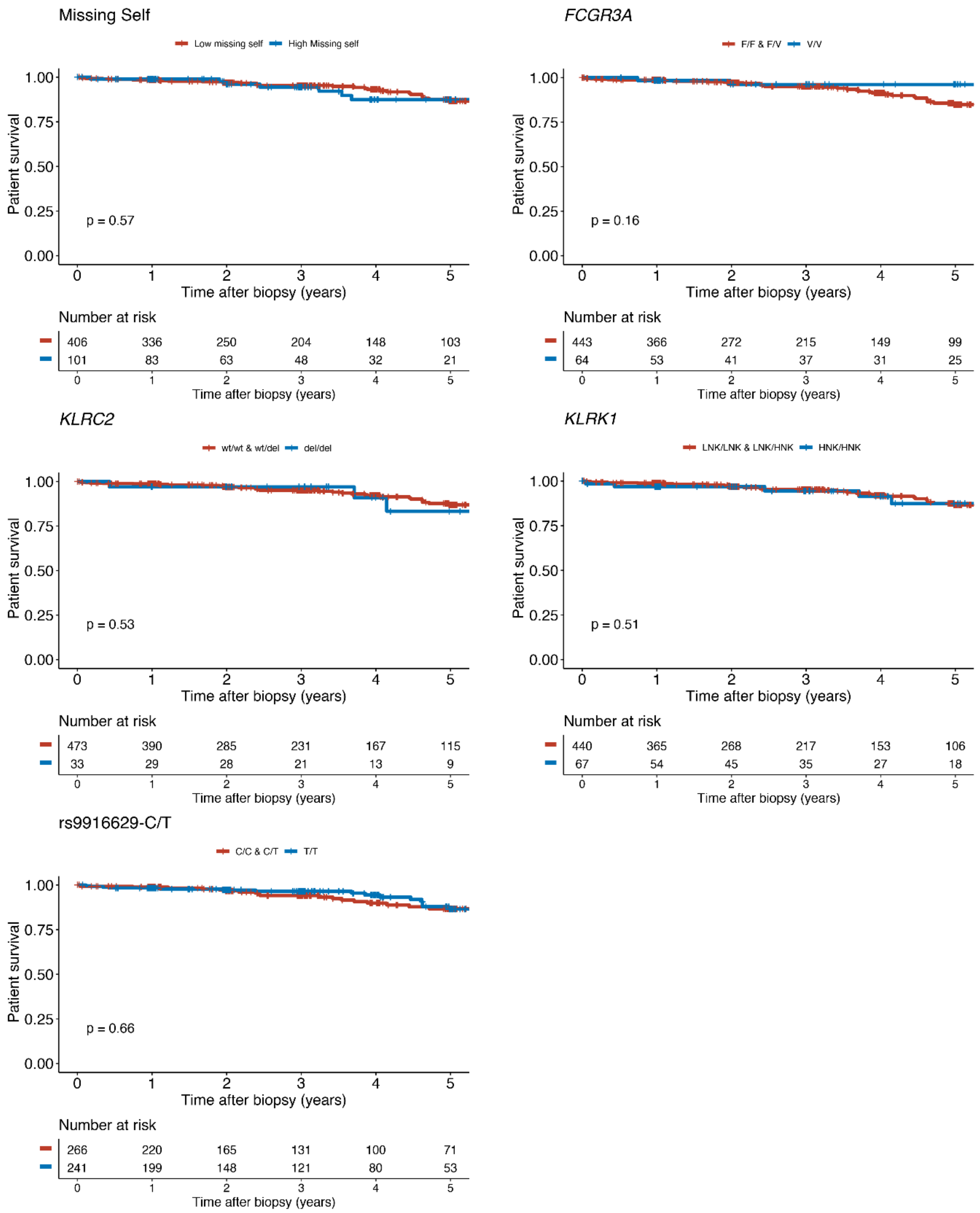

For these analyses, missing self was dichotomized into high missing self (≥2) and low missing self (< 2). The genetic polymorphisms were also dichotomized as described in the methods section. P-value is calculated with a log-rank test.

Figure S4: Distribution of MVI among genetic variants and missing self, stratified by CMV viremia

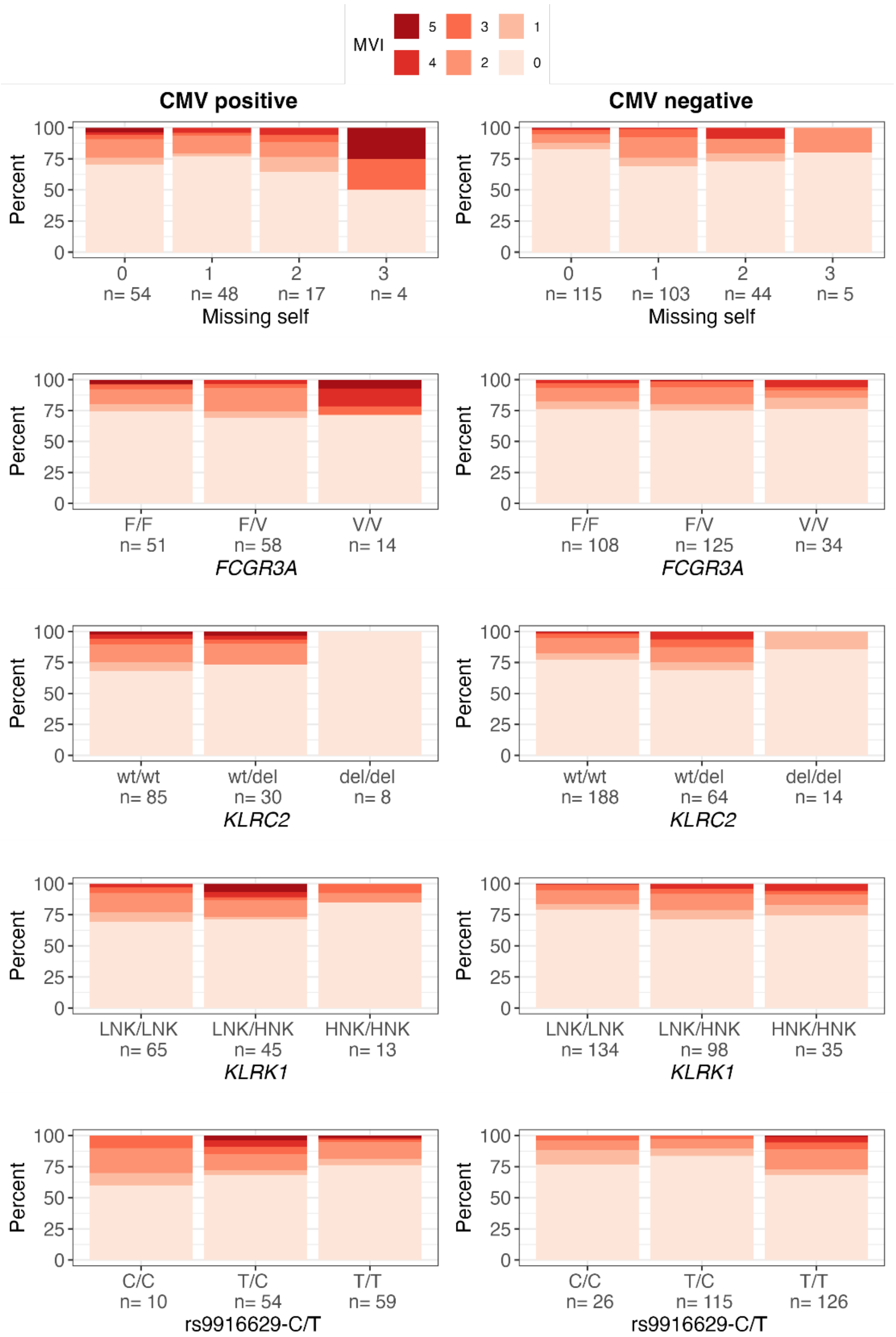

The bars represent subjects stratified by individual genotypes or the number of missing self, specifically for patients with a positive cytomegalovirus PCR result. Proportions of individual MVI scores are indicated by different shades of red. CMV viremia was missing in 76 patients (15%). Overall, 133 (26.2%) patients had a positive CMV PCR results after transplantation and

CMV viremia was equally distributed among patients that developed MVI (n=25, 36.2%) and patients that did not develop MVI (n=98, 25.1%, p=0.170).
Abbreviations: MVI, microvascular inflammation; PCR, Polymerase Chain Reaction.

Figure S5: Distribution of MVI among genetic variants and missing self, stratified by donor type

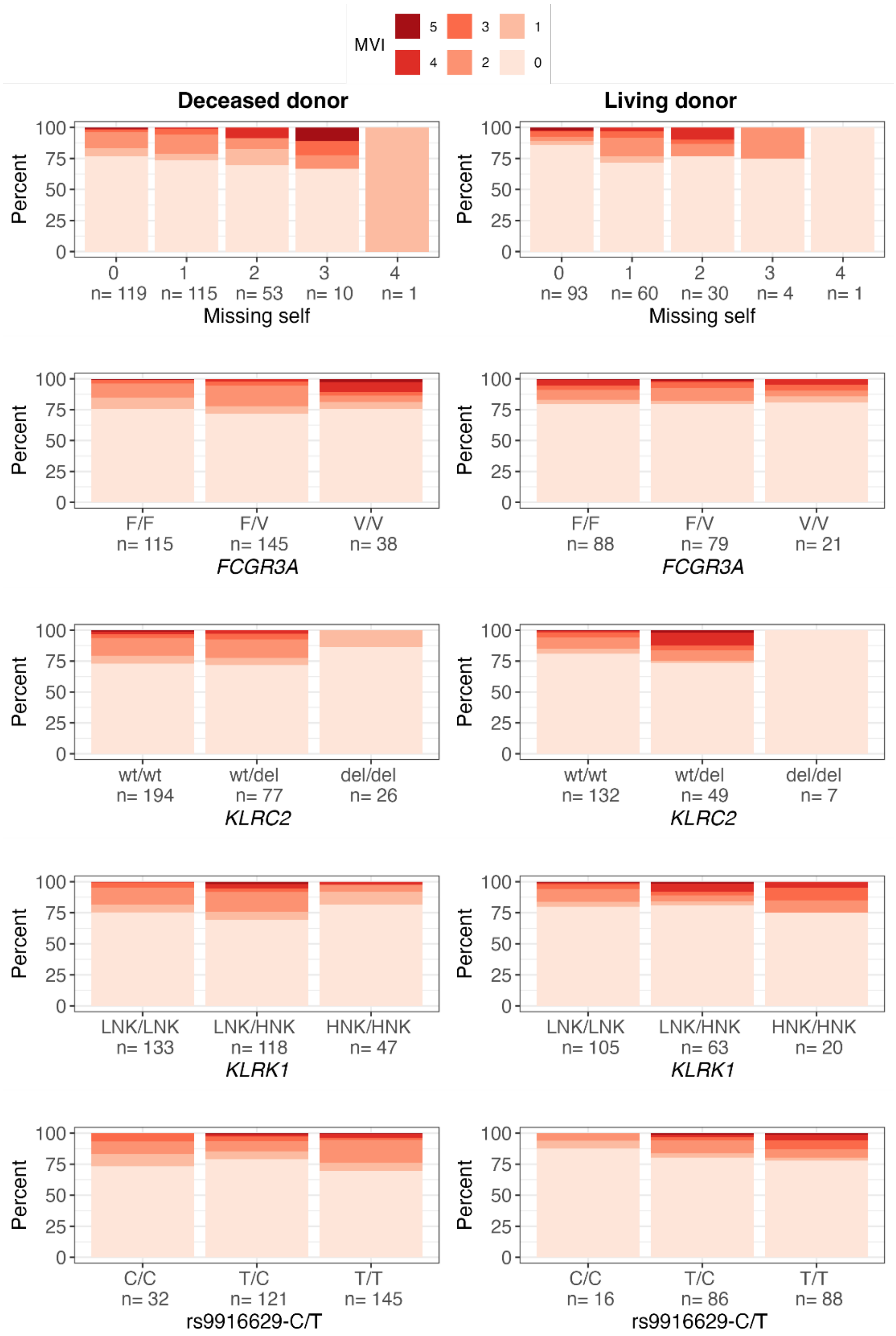

The bars represent subjects stratified by individual genotypes or the number of missing self, further stratified by the type of donation. Proportions of individual MVI scores are indicated by different shades of red.

Abbreviations: MVI, microvascular inflammation.

Figure S6 Distribution of MVI among genetic variants and missing self, stratified by cold ischemia time

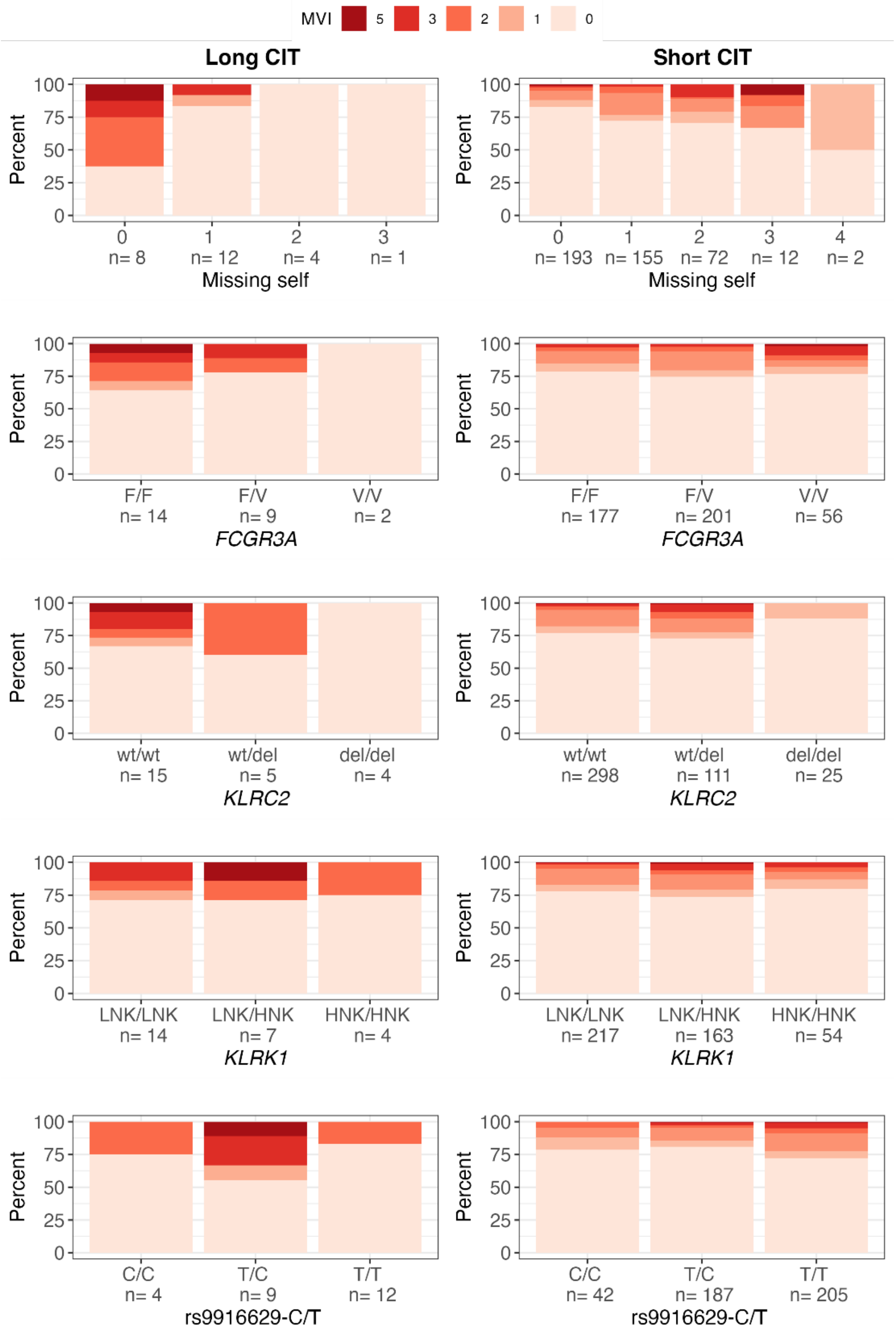

The bars represent subjects stratified by individual genotypes or the number of missing self, further stratified by the cold ischemia time (CIT). Cold ischemia time was defined long (>16 hours) and short (<16 hours). Proportions of individual MVI scores are indicated by different shades of red.

Abbreviations: MVI, microvascular inflammation.

Table S1. Brant-Wald Test.

Variable	chi^2	Degrees of freedom	*P*-value
**All**	44.08	40	0.30
**Missing self**	2.15	4	0.71
KLRC2	0	4	>0.99
KLRK1	0.18	4	>0.99
FCGR3A	6.7	4	0.15
**rs9916629-C/T**	1.12	4	0.89
**DSA**	4.91	4	0.30
**Eplet count (HLA-DR)**	5.53	4	0.24
**Eplet count (HLA-DQ)**	6.49	4	0.17
**Eplet count (HLA-DP)**	2.89	4	0.58
**Number of biopsies**	12.06	4	0.02

Table S2. Hardy Weinberg equilibrium

Gene	Chi-square	*P*-value
FCG3A	0.014	0.905
KLRC2	15.928	<0.001
KLRK1	10.129	0.001
**rs9916629-C/T**	0.090	0.765

Table S3: Mixed effects proportional odds regression model for MVI.

Variants	Multivariable	
	OR (95%CI)	*P*-value
Missing self	1.48 (0.97 to 2.27)	0.071
FCGR3A$^{V/V158}$	1.01 (0.33 to 3.07)	0.986
KLRC2$^{del/del}$	0.12 (0.01 to 1.02)	0.052
KLRK1$^{HNK/HNK}$	1.36 (0.36 to 5.07)	0.652
rs9916629 T/T	1.77 (0.85 to 3.68)	0.124

Abbreviations: CI, confidence interval; OR, odds ratio. The model included time since transplantation as fixed effect and a random intercept for patients.

Table S4: **Causes for graft loss in patients with high missing self types (>=2).**

Final adjusted reason for graft loss	Banff 2019 diagnosis	Pretransplant risk	MVI
Chronic active ABMR	Active ABMR	Preformed DSA	g1, ptc3
C4d - DSA- MVI with severe transplant glomerulopathy.	No rejection	Husband to wife with 2 shared children; no DSA	g2, ptc3
Multifactorial. Poor organ quality with delayed graft function and tubular damage. Morphology: CNI toxicity and later, two episodes of rejection	Borderline lesion	None	g0, ptc0
Active ABMR	Active ABMR	Preformed DSA	g2, ptc2
Poor kidney quality with marginal graft function for only a few weeks.	TCMR IIB	None	g0, ptc0
Poor organ quality. Delayed graft function over 3 weeks post-transplantation. Sudden bleeding from the renal artery. The kidney had to be removed.	No rejection	None	g0, ptc0
Primary non-function in the presence of severe hypotension in a patient with amyloidosis and a donor organ with hypertensive predamage. Allograft biopsies revealed no rejection, but persistent irreversible ischemic damage.	TCMR, Banff type IIA	None	g0, ptc0
A case of multifactorial transplant failure: Initially, the patient was diagnosed with TCMR. Subsequently, infectious complications and severe diarrhea led to acute kidney failure. A re-biopsy revealed tubular damage and oxalosis.	TCMR, Banff type IA	Repeated mismatch	g0, ptc0

Severe vascular rejection with rapid progression to terminal graft failure, despite early anti-rejection therapy.	Banff type III rejection	None	g0, ptc0
A case of multifactorial transplant failure: primarily irreversible ischemic graft damage. DCD kidney with prolonged ischemia following bleeding under ATG administration, followed by vascular rejection on postoperative day 7.	TCMR IIA	None	g0, ptc0

The final adjusted diagnosis was defined by nephrologists, taking into account all clinical and laboratory data. In cases of multiple biopsies, the highest rejection grade according to the Banff classification was documented.

Abbreviations: ABMR, antibody-mediated rejection; CNI, calcineurin inhibitor; DCD, donor after cardiac death; DSA, donor-specific antibodies; g, glomerulitis; MVI, microvascular inflammation; ptc, peritubular capillaritis; TCMR, T-cell-mediated rejection.

Table S5: Trajectories of eGFR in relation to missing self and gene variants.

Variable		Estimated eGFR slope (95% CI)	*P*-Value
**Missing self**			
**First year**	Low missing self	4.92 (3.58 to 6.27)	
	High Missing self	6.01 (3.28 to 8.73)	
	Inter-group difference	1.08 (-1.96 to 4.12)	0.4855
**After first year**	Low missing self	-0.50 (-1.08 to 0.08)	
	High missing self	-2.42 (-6.71 to 1.87)	
	Inter-group difference	-0.26 (-1.59 to 1.07)	0.7046
**Total**	Low missing self	0.84 (0.32 to 1.36)	
	High missing self	0.91 (-0.15 to 1.97)	
	Inter-group difference	0.07 (-1.11 to 1.25)	0.9040
FCGR3A			
**First year**	F/F & F/V	5.24 (3.94 to 6.53)	
	V/V	4.47 (1.14 to 7.81)	
	Inter-group difference	-0.76 (-4.34 to 2.81)	0.6750
**After first year**	F/F & F/V	-0.58 (-1.14 to -0.02)	
	V/V	1.53 (-3.55 to 6.62)	
	Inter-group difference	0.22 (-1.24 to 1.67)	0.7691
**Total**	F/F & F/V	0.85 (0.35 to 1.35)	
	V/V	0.83 (-0.43 to 2.09)	
	Inter-group difference	-0.02 (-1.38 to 1.33)	0.9723
KLRC2			
**First year**	wt/wt & wt/del	5.34 (4.09 to 6.59)	
	del/del	2.60 (-2.02 to 7.21)	
	Inter-group difference	-2.74 (-7.52 to 2.03)	0.2605
**After first year**	wt/wt & wt/del	-0.64 (-1.18 to -0.10)	
	del/del	-9.23 (-16.04 to -2.42)	
	Inter-group difference	1.16 (-0.79 to 3.11)	0.2436
**Total**	wt/wt & wt/del	0.83 (0.35 to 1.32)	

	del/del	1.03 (-0.66 to 2.72)	
	Inter-group difference	0.20 (-1.56 to 1.95)	0.8248
KLRK1			
**First year**	LNK/LNK & HNK/LNK	5.00 (3.71 to 6.29)	
	LNK/LNK	6.08 (2.72 to 9.45)	
	Inter-group difference	1.08 (-2.53 to 4.68)	0.558
**After first year**	LNK/LNK & HNK/LNK	-0.70 (-1.25 to -0.14)	
	HNK/HNK	-3.04 (-8.13 to 2.05)	
	Inter-group difference	1.07 (-0.44 to 2.59)	0.166
**Total**	LNK/LNK & HNK/LNK	0.71 (0.21 to 1.21)	
	HNK/HNK	1.78 (0.50 to 3.06)	
	Inter-group difference	1.07 (-0.30 to 2.45)	0.125
**rs9916629-C/T**			
**First year**	T/T & T/C	5.14 (3.47 to 6.80)	
	C/C	5.16 (3.41 to 6.90)	
	Inter-group difference	0.02 (-2.39 to 2.43)	0.9861
**After first year**	T/T & T/C	-0.20 (-0.92 to 0.51)	
	C/C	1.09 (-2.35 to 4.53)	
	Inter-group difference	-0.73 (-1.77 to 0.30)	0.1656
**Total**	T/T & T/C	1.11 (0.47 to 1.76)	
	C/C	0.56 (-0.11 to 1.23)	
	Inter-group difference	-0.55 (-1.48 to 0.38)	0.2470

The Chronic Kidney Disease Epidemiology Collaboration (CKD-EPI 2009) estimated glomerular filtration rate (eGFR) slope was estimated using a linear mixed-effects model beginning three months after transplantation using an unstructured covariance-variance matrix. Considering the natural trajectory of kidney function after transplantation we used a knot after one year. The total slope was calculated as a linear contrast of the slope in the first year and the years afterwards. The model included fixed effect of the polymorphism, time, a spline term and the two-way interaction of polymorphism and time and polymorphism and spline.

Abbreviations: eGFR, estimated glomerular filtration rate; low missing self, missing self <2; high missing self, missing self ≥2.

Table S6: Proportional odds regression model in patients with preformed or *de novo* DSA (n=86).

Variants	Univariable	
	OR (95%CI)	*P*-value
Missing self	1.24 (0.69 to 2.23)	0.459
FCGR3A$^{V/V158}$	4.14 (0.99 to 17.47)	0.052
KLRC2$^{del/del}$	0.63 (0.03 to 5.21)	0.686
KLRK1$^{HNK/HNK}$	1.38 (0.33 to 5.07)	0.636
rs9916629 T/T	2.56 (1.02 to 6.66)	0.046

Abbreviations: CI, confidence interval; OR, odds ratio.
